# Serology describes a profile of declining malaria transmission in Farafenni, The Gambia

**DOI:** 10.1186/s12936-015-0939-1

**Published:** 2015-10-22

**Authors:** Lotus L. van den Hoogen, Jamie T. Griffin, Jackie Cook, Nuno Sepúlveda, Patrick Corran, David J. Conway, Paul Milligan, Muna Affara, Stephen J. Allen, Carla Proietti, Serign J. Ceesay, Geoffrey A. Targett, Umberto D’Alessandro, Brian Greenwood, Eleanor M. Riley, Chris Drakeley

**Affiliations:** Department of Immunology and Infection, Faculty of Infectious and Tropical Diseases, London School of Hygiene and Tropical Medicine, Keppel Street, London, WC1E 7HT UK; MRC Centre for Outbreak Analysis and Modelling, Department of Infectious Disease Epidemiology, Faculty of Medicine, Imperial College London, London, W2 1PG UK; Centre of Statistics and Applications of University of Lisbon, Faculdade de Ciências Da Universidade de Lisboa, Bloco C6—Piso 4, 1749-1016 Lisbon, Portugal; Disease Control and Elimination, Medical Research Council Unit, Fajara, 220 The Gambia; Department of Clinical Sciences, Liverpool School of Tropical Medicine, Liverpool, L3 5QA UK; Infectious Diseases Programme, QIMR Berghofer Medical Research Institute, Brisbane, QLD 4029 Australia

**Keywords:** *Plasmodium falciparum*, Malaria, Transmission, Serology, MSP-1_19_

## Abstract

**Background:**

Malaria morbidity and mortality has declined in recent years in a number of settings. The ability to describe changes in malaria transmission associated with these declines is important in terms of assessing the potential effects of control interventions, and for monitoring and evaluation purposes.

**Methods:**

Data from five cross-sectional surveys conducted in Farafenni and surrounding villages on the north bank of River Gambia between 1988 and 2011 were compiled. Antibody responses to MSP-1_19_ were measured in samples from all surveys, data were normalized and expressed as seroprevalence and seroconversion rates (SCR) using different mathematical models.

**Results:**

Results showed declines in serological metrics with seroprevalence in children aged one to 5 years dropping from 19 % (95 % CI 15–23 %) in 1988 to 1 % (0–2 %) in 2011 (*p* value for trend in proportions < 0.001) and the SCR dropping from 0.069 year^−1^ (0.059–0.080) to 0.022 year^−1^ (0.017–0.028; p = 0.004). The serological data were consistent with previously described drops in both parasite prevalence in children aged 1–5 years (62 %, 57–66 %, in 1988 to 2 %, 0–4 %, in 2011; p < 0.001), and all-cause under five mortality rates (37 per 1000 person-years, 34–41, in 1990 to 17, 15–19, in 2006; p = 0.059).

**Conclusions:**

This analysis shows accurate reconstruction of historical malaria transmission patterns in the Farafenni area using anti-malarial antibody responses. Demonstrating congruence between serological measures, and conventional clinical and parasitological measures suggests broader utility for serology in monitoring and evaluation of malaria transmission.

**Electronic supplementary material:**

The online version of this article (doi:10.1186/s12936-015-0939-1) contains supplementary material, which is available to authorized users.

## Background

Monitoring changes in malaria transmission levels due to natural fluctuations or as a result of control programmes is a key component of disease surveillance. There are a number of metrics available to assess malaria transmission ranging from passively collected morbidity and mortality data at health facilities to active detection of parasites in the mosquito and human host from field studies. The utility of different measures depends on a variety of factors including the capacity of the health system to collect and analyse routine clinical data, local ecology of vectors and transmission intensity itself [1, 2].

Serological measures based on human anti-malarial antibodies can be used to assess malaria transmission intensity. Antibodies are produced during an infection with the *Plasmodium* parasite and are boosted upon subsequent infections. The presence of *Plasmodium*-specific antibody levels reflects exposure and, since many antibody responses are longer lived than infections in humans, they represent cumulative exposure over time. Therefore, anti-malarial antibody responses represent a population’s history of malaria transmission [3]. Antibody responses can be expressed as age-adjusted continuous or binary variables to generate a seroconversion rate (SCR) which is analogous to the force of infection [3]. The SCR reflects the rate at which individuals become seropositive and can be estimated using simple reversible catalytic [3] or superinfection models [4]. The SCR has been used to describe malaria transmission intensity [3, 5], as well as to measure a change in malaria transmission [5–8] and to assess the effects of malaria control measures on transmission [7]. Serological measures are useful additional metrics to measure malaria transmission intensity, alongside parasite prevalence (parasite rate, PR) in humans and the entomological inoculation rate (EIR), especially at low transmission levels where other metrics lose their ability to discriminate [1, 9].

There are relatively few examples of repeated surveys to examine the validity of serological estimates of transmission over time. Historically, malaria transmission has been well-described in The Gambia with significant declines in malaria indices over the last 25 years [10–13]. Due to a longstanding collaboration between the London School of Hygiene and Tropical Medicine (LSHTM) and the Medical Research Council (MRC) in The Gambia, seroprevalence and PR data, obtained from samples collected in the villages around Farafenni in The Gambia, were combined. In Farafenni, on the north bank of the River Gambia, malaria was hyperendemic in 1988 with a PR over 60 % in children under 15 years of age [14, 15]. However, overall dry season microscopy PRs declined from 29 % in 1988 [14] to 11 % in 2008 [16]. In order to assess the utility of serological measures in describing this decline of malaria transmission, seroprevalence data from the Farafenni area between 1988 and 2011 have been compared with PR and previously described mortality rates.

## Methods

### Study area

**Fig. 1:**
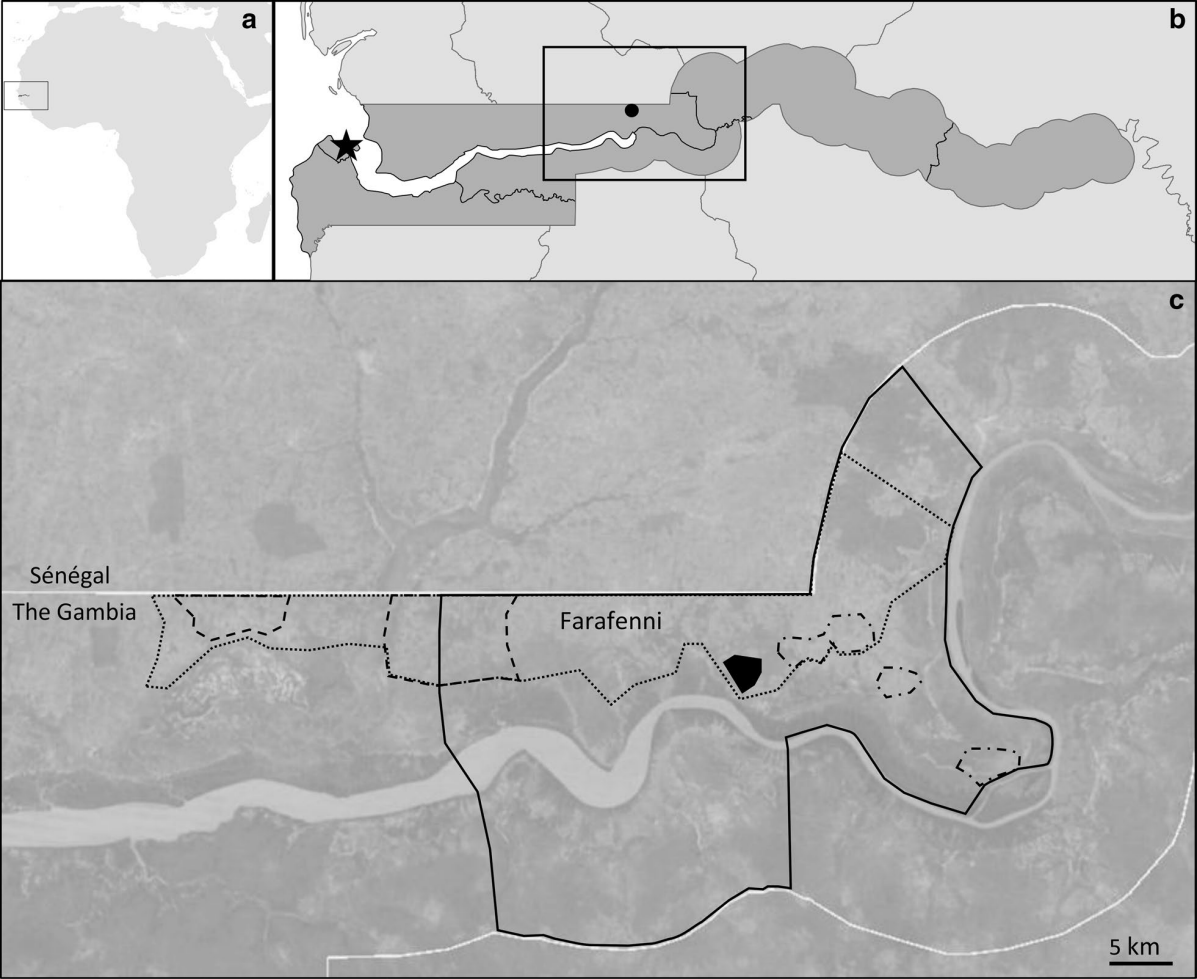
Map of **a** Africa, **b** The Gambia and **c** the Farafenni area. In **b** the *star* represents the capital Banjul and the *solid dot* represents Farafenni. In **c** the location of the five surveys was as follows: 1988 Kataba hamlets (*solid black fill*), 1990 Fula hamlets and surrounding villages (*dashed lines*), 2003 the catchment area of the Farafenni hospital (*solid black line*), 2008 Dibba Kunda, Bambali, Sara Kunda and Pallen Wollof (*dotted–dashed lines*) and 2011 the Farafenni area (*dotted line*)

Farafenni is situated on the north bank of the River Gambia, approximately 3 km from the border with Senegal (Fig. 1). Malaria transmission in the area is highly seasonal with nearly all transmission occurring during and after the rainy season [14], characterized by intense rains from June to October, with transmission continuing until December [10, 17]. *Plasmodium falciparum* is responsible for nearly all malaria infections in Farafenni [14, 18].

### Data collection and laboratory methods

Original datasets from surveys in 1988, 1990, 2003, 2008, and 2011, conducted as part of a longstanding collaboration between the LSHTM and the MRC in The Gambia, were obtained (Table 1). The location of the participating villages in the Farafenni area during these studies is shown in Fig. 1. A full description of the study population, data collection and laboratory methods for each survey is detailed elsewhere [14, 16, 19–23].

**Table 1:** General characteristics of the surveyed study population between 1988 and 2011 in Farafenni, the Gambia

% (95 % CI)	1988	1990	2003	2008	2011
Timing of survey	November	November	July^a^	January/february	October
Season	Peak transmission	Peak transmission	Start wet season	Dry season	Peak transmission
Median age in years (range)	5 (1–41)	14 (1–90)	11 (1–83)	13 (1–80)	10 (1–15)
Parasite prevalence^b^					
1–5 year olds	62 (57–66)	17 (11–23)	39^c^ (31–46)	16 (9–22)	2 (0–4)
1–15 year olds	60 (56–64)	22 (18–26)	40^d^ (33–46)	18 (14–22)	3 (1–4)
All ages	53 (49–57)	16 (13–19)	34 (30–38)	13 (11–16)	3 (1–4)
Number sampled for serology	742	736	624	670	488
Seroprevalence^e^					
1–5 year olds	19 (15–23)	19 (12–25)	12 (7–17)	13 (7–19)	1 (0–2)
1–15 year olds	23 (20–26)	31 (27–36)	23 (18–27)	20 (16–24)	13 (10–16)
All ages	28 (25–31)	46 (42–50)	35 (31–39)	34 (30–38)	13 (10–16)
Reference	[14, 19]	[20, 21]	[22]	[16]	[23]

For 2011 ‘all ages’ only consists of children up to the age of 15 years

*CI* confidence interval

^a^Survey for parasite prevalence performed in November

^b^Parasite prevalence for *P. falciparum* as measured by polymerase chain reaction in the 2011 survey and microscopy of blood films in all other surveys

^c^For age 6 months–5 years

^d^For age 6 months–15 years

^e^Seroprevalence for MSP-1_19_

The enzyme-linked immunosorbent assay (ELISA) protocol was the same in all surveys [24] and antibody responses to the 19 Kda fragment of *P. falciparum* merozoite surface protein 1 (MSP-1_19_) were recorded together with demographic data (e.g., age, sex) and parasite status. ELISAs were performed in The Gambia for all surveys, except 1990.

### Statistical analyses

All statistical analyses were conducted in Stata (version 13.1) and Prism (version 6.02). Infants under 1 year of age were excluded from each dataset to remove any influence of maternally derived antibodies [3]. A two-component Gaussian mixture model [25] was used to determine separate cut-off values for seropositivity for each dataset. SCRs (i.e., the rate at which the population becomes seropositive) and seroreversion rates (SRR; i.e., the rate at which the population reverts back to being seronegative) were calculated by fitting four different models. Firstly, a simple reversible catalytic model was fitted to age-adjusted seropositivity data using maximum likelihood [3]. Secondly, data were fitted to a superinfection model [4] which is comparable to the reversible catalytic model but allows for prolonged periods in the seropositivity state due to recurrent malaria exposure. More precisely, this model assumes that when an individual is re-infected while seropositive, the antibody response can be boosted and thus individuals can move between multiple seropositive states. Fixing and allowing SRR to vary was investigated for both of these models; the common SRR was estimated using maximum likelihood as described previously [3]. Furthermore, the presence of specific change points in transmission was investigated for each of the surveys separately as previously described [5, 7]. A likelihood ratio test was used to determine whether a model with a change point in transmission fit the data better than a model using one SCR. Finally, the change in the SCR was also investigated using an extension of the reversible catalytic model that allows multiple changes in the SCR, which was fitted to all surveys simultaneously. This novel approach can estimate the parameters using penalized maximum likelihood, by assuming that the log of the ratio of successive SCRs follows a normal distribution. This assumption results in smoothed estimates of the SCR over time, and so the model is referred to here as the ‘smoothed model’. The reversible catalytic models are implemented as a Stata program called revcat (see Additional file 1) [26].

Chi-squared was used to test differences between proportions from two surveys while the Wilcoxon–Mann–Whitney test was applied to continuous outcomes to compare the medians of two independent surveys. The trend in previously published all-cause under five-year-old mortality rates per 1000 person-years (U5MR) for the Farafenni area and the SCR estimates over time were calculated with linear regression. The Cochrane-Armitage test for trend in proportions was used to test the trend in the PR and seroprevalence. PR, U5MR, SRR, as well as SCR estimates, are followed by 95 % confidence intervals in brackets.

## Results

The number of individuals sampled in each survey ranged from 488 in 2011 to 742 in 1988, Table 1. The 1988, 1990 and 2011 surveys were performed during the peak transmission season (October/November), while the 2003 survey was conducted during the start of the wet season (July) and the 2008 survey during the early dry season (January/February). The median age and age range were comparable in the 1990–2008 surveys. However, the median age was lower in the 1988 survey and the age range was lower in the 2011 survey, which only included children up to the age of 15 years. Seroprevalence to MSP-1_19_ in 1–5 year olds decreased from 19 % (15–23 %) in 1988 to 1 % (0–2 %) in 2011 (p < 0.001), while the PR in 1–5 year olds declined from 62 % (57–66 %) in 1988 to 2 % (0–4 %) in 2011 (p < 0.001). When comparing the 1988 and 1990 surveys, overall seroprevalence was lower (28 vs 46 %, p < 0.001) and human parasite carriage was higher (53 vs 16 %, p < 0.001) in 1988, even though both surveys were performed in the same season and were only 2 years apart. A plausible explanation for this could be the younger population in 1988 compared to 1990 (median age 5 vs 14 years, p < 0.001).

**Fig. 2:**
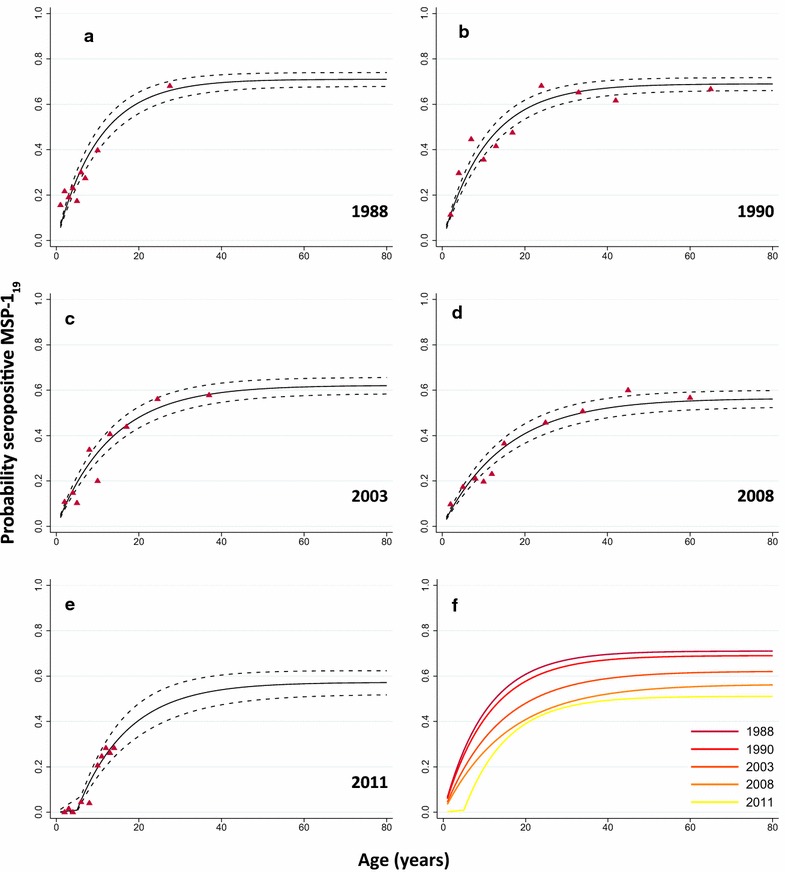
Predicted age-seroprevalence curves for MSP-1_19_ between 1988 and 2011 in Farafenni, The Gambia. MSP-1_19_ seroconversion curves per survey using the reversible catalytic model (with a fixed common seroreversion rate across all five surveys: 0.028 year^−1^). In the separate graphs for the five surveys (**a**–**e**) *solid lines* represent the fitted probability for being seropositive to MSP-1_19_, *dotted lines* represent the 95 % confidence interval for these fits and *triangle*s represent the observed proportion of seropositives per age decile. The five separate fits are combined in graph (**f)**

**Fig. 3:**
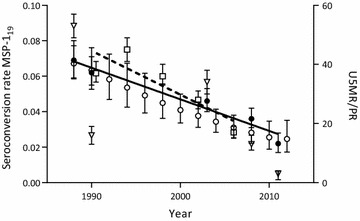
Trend in the seroconversion rate, all-cause mortality rate and parasite prevalence in Farafenni, The Gambia. Linear regression of MSP-1_19_ seroconversion rate (SCR) estimates per survey using the reversible catalytic model (with a fixed common seroreversion rate across all five surveys: 0.028 year^−1^; *solid circles* and *solid line*), SCR estimates per 2 years using the smoothed model (*open circles*), linear regression of all-cause under 5-year-old mortality rate per 1000 person-years (U5MR) as previously published by Jasseh et al. [28] (*squares* and *dashed line*), and parasite prevalence (PR) per survey (as measured by polymerase chain reaction in the 2011 survey and microscopy of blood films in all other surveys; *triangles*). Survey data are from five surveys in the Farafenni area between 1988 and 2011

The reversible catalytic and superinfection models led to similar results with an overall decrease in the SCR between 1988 and 2011 of 67–68 % when the SRR was fixed and 74–76 % when the SRR was allowed to vary, Table 2. Since both models provided similar results, the reversible catalytic model with a fixed SRR was used for further analysis. Predicted age seroprevalence curves for MSP-1_19_ for each survey are shown in Fig. 2. The SCR declined from 0.069 year^−1^ (0.059–0.080) in 1988 to 0.022 year^−1^ (0.017–0.028) in 2011 (p = 0.004), Fig. 3. The SCR values suggest a drop in the estimated annual EIR from ~5 to ~0.5 infectious bites per person per year (ib/p/y) using the previously described relationship between SCR and EIR [3, 27]. The previously published U5MR in the Farafenni area [28] showed an overall decline of 54 %; from 37 per 1000 person-years (34–41) in 1990 to 17 (15–19) in 2006 (p = 0.059; Fig. 3).

**Table 2:** Seroconversion and seroreversion rate estimates for MSP-1_19_ between 1988 and 2011 in Farafenni, the Gambia

Year	Reversible catalytic model							Superinfection model						
	Varied SRR (average SRR 0.024 year^−1^; time to become seronegative 42 years)				Fixed SRR (common SRR 0.028 year^−1^; time to become seronegative 36 years)			Varied SRR (average SRR 0.043 year^−1^; time to become seronegative 23 years)				Fixed SRR (common SRR 0.049 year^−1^; time to become seronegative 20 years)		
	SRR (95 % CI)	SCR (95 % CI)	% Decrease in SCR since previous survey	% Decrease in SCR since 1988 survey	SCR (95 % CI)	% Decrease in SCR since previous survey	% Decrease in SCR since 1988 survey	SRR (95 % CI)	SCR (95 % CI)	% Decrease in SCR since previous survey	% Decrease in SCR since 1988 survey	SCR (95 % CI)	% Decrease in SCR since previous survey	% Decrease in SCR since 1988 survey
1988	0.039 (0.017–0.090)	0.073 (0.059–0.091)			0.069 (0.059–0.080)			0.070 (0.035–0.137)	0.078 (0.062–0.099)			0.072 (0.062–0.083)		
1990	0.037 (0.023–0.059)	0.070 (0.055–0.088)	4	4	0.062 (0.055–0.071)	10	10	0.071 (0.047–0.106)	0.078 (0.060–0.100)	0	0	0.064 (0.046–0.091)	11	11
2003	0.022 (0.011–0.045)	0.043 (0.034–0.055)	39	41	0.046 (0.040–0.053)	26	33	0.038 (0.020–0.073)	0.046 (0.035–0.060)	41	41	0.050 0.036–0.072	22	31
2008	0.022 (0.011–0.041)	0.033 (0.026–0.043)	23	55	0.036 (0.031–0.042)	22	48	0.036 (0.020–0.064)	0.036 (0.027–0.047)	22	54	0.041 (0.029–0.059)	18	43
2011	~0 (NA)	0.019 (0.015–0.024)	42	74	0.022 (0.017–0.028)	39	68	~0 (NA)	0.019 (0.015–0.024)	47	76	0.024 (0.016–0.037)	42	67

*CI* confidence interval; *NA* not applicable. Seroconversion rate (SCR) and seroreversion rate (SRR) estimates according to the reversible catalytic and superinfection model for five surveys between 1988 and 2011, while fixing the SRR and allowing the SRR to vary

When the SRR was allowed to vary across surveys it was between 1.7 and 1.9 times as high in the surveys in 1988 and 1990 in comparison with the more recent surveys in 2003 and 2008 (Table 2). Since only individuals under 15 years old were included in 2011, the SRR for that year approximated to zero. The SRRs reflect the time for a seropositive individual to become seronegative, which ranged from ~14–26 years in the earlier surveys (superinfection model and reversible catalytic model, respectively) to ~27–46 years in the more recent surveys.

Analysis per survey—while fixing the SRR to the previously determined common SRR (0.028 year^−1^)—identified a significant change in transmission intensity only in the 2011 survey data and indicated that the change occurred in 2006 (5 years previously; range 4–8; p < 0.001). A corresponding change point was not seen in the 2008 survey (p = 0.179). SCR estimates for the 2011 surveys were as follows: 0.002 year^−1^ (0.000–0.013) up to the age of 5 and 0.053 year^−1^ (0.040-0.070) for those aged five and over (Fig. 2e). The SCR before the change point reflects most recent transmission and suggests an even more pronounced decline in the SCR of 97 % since 1988. However, since this survey only included children, it was decided to use the ‘average’ SCR for 2011 in the analyses described above.

The smoothed model estimating a separate SCR for each two-year period between 1988 and 2012 produced similar results to the fixed reversible catalytic model as shown in Fig. 3. The SCR declined from 0.067 year^−1^ (0.059–0.077) in 1988 to 0.025 year^−1^ (0.017–0.035) in 2012; reflecting an overall decrease of 63 % (46–75 %). The common SRR in the smoothed model was 0.033 year^−1^ (0.026–0.043), equating to an antibody decay time of 30 years.

## Discussion

This study describes the retrospective analysis of antibody responses to the MSP-1_19_ antigen of *P. falciparum* malaria in samples collected over a 23-year period in villages in the Farafenni area, The Gambia. Serological outcomes showed a clear relation to documented changes in malaria transmission with a decline in the seroprevalence and the SCR in line with a decline in parasite prevalence and all-cause mortality rates in children under 5 years old (U5MR).

The data describe very similar decreases over the time period in parasite prevalence (from 62 to 2–97 % decrease) and seroprevalence (from 19 to 1–95 % decrease) in children under 5 years old, while other studies from this area [23, 25] and elsewhere [29] have typically observed serological measures to be consistently higher than infection measures. Although similar prevalence of parasite carriage by PCR and seropositivity has been previously described in the Farafenni area [16], the reasons for the results in the current study are unclear. It may be due to the fact that responses to only one malaria antigen were assessed and, therefore, the data represent a minimal estimate of seroprevalence. All age seroprevalence predictably is higher than infection measures and operationally it may be logistically more attractive to focus on the whole population rather than specific age groups to generate these data. Further analysis is underway to examine parasite and serological metrics over a much wider geographical range.

Analysis of age-related seroprevalence data collected using both the reversible catalytic [3] and the superinfection model [4] gave similar estimates for seroconversion irrespective of whether the SRR was fixed or allowed to vary across surveys [3]. Estimates from different models suggest a decline in seroconversion of approximately 70 % between 1988 and 2011, equating to an estimated ten-fold reduction in EIR to less than 1 ib/p/year. This is consistent with reports of declines in transmission both in The Gambia [12, 13], including Farafenni [10, 13], based on a variety of metrics (PR and U5MR), and similar declines between 1990 and 2012 reported by Trape et al. in nearby Dielmo, Senegal [30]. The reported decline in malaria transmission in Farafenni is likely related to scale-up of national intervention policies during this period (free malaria diagnosis in 1998, the distribution of free bed nets in 2000, intermittent preventive treatment in pregnant women in 2002, and the authorization of DDT for indoor residual spraying in 2007) [11]. Whilst caution should be observed with any extrapolation, data from the linear trend suggest a predicted current SCR for 2015 of 0.020 year^−1^ (0.010–0.030) which correlates with an estimated EIR of ~0.4 ib/p/year.

Previous serological analyses of samples from Bioko Island, Equatorial Guinea [7] and southern Vanuatu [6] have shown a step-change in malaria transmission representing a distinct drop in exposure to infection after the introduction of malaria control methods. This is a unique facet of serological measures in that, when integrated with age, they allow examination of historical changes in exposure to infection. In the current analysis the absence of a step phenomenon in the smoothed model, and only in the 2011 survey when surveys were analysed separately, indicates that transmission reduction has been gradual rather than sudden. This may reflect the steady increase in intervention delivery, coverage and uptake as illustrated in the country profile in the WHO World Malaria Report 2014 [11].

The absence of a step-change (before the 2011 survey) may also be due to the sample sizes being too small to detect this phenomenon [31]. A recent serological analysis of a longitudinal sample set from Asembo Bay in Kenya similarly did not find a distinct drop in transmission associated with the widespread distribution of nets in the 1990s despite significant reductions in morbidity and mortality at the time. This was most likely due to a combination of limited sample size and a gradual decrease in transmission [32]. In the current analysis the change point in 2006 (detected in the 2011 survey) corresponds with previously reported minimal MSP-1_19_ responses under the age of ten in 2009 by Ceesay et al. [13]. It suggests an even more pronounced decline in the SCR of 97 % since 1988. However, the 2011 data presented here included children only up to the age of 15 years and it would have been interesting to analyse the full serological profile across all ages to confirm this trend.

Interestingly, when the SRR was allowed to vary across surveys, it was nearly two-fold higher in the older surveys (1988 and 1990) compared to the more recent surveys (2003 and 2008), suggesting slightly paradoxically that the average decay time for antibodies increased while malaria transmission decreased. This increase in the half-life of anti-malarial antibodies over time might be explained by the fact that fewer children are infected in recent surveys and thus older individuals with more established antibody responses remain. It has previously been observed that anti-malarial antibody titres are relatively stable (consisting of long-lived plasma cells after repeated exposure) in adults but very variable (consisting of short-lived plasma cells) in children [33].

As previously described by Bosomprah et al. [4] the superinfection and reversible catalytic models gave very similar estimations of the SCR, yet the time for anti-malarial antibodies to decay was approximately 1.8 times faster in the superinfection model. This gave more realistic estimations of the time to decay (approximately 20 years) in comparison with the previously described minimum of 10 years [3]. Although both findings, the increased half-life of anti-malarial antibodies following declining transmission as well as the overall decreased half-life in the superinfection model, seem valid, further research is still needed to assess antibody decay rates in relation to age.

The reversible catalytic model was extended to a model that allowed fitting all surveys simultaneously and to allow multiple SCRs, with a smoothing parameter to prevent over-fitting. This model is particularly useful when the trend in the SCR, with a possible change point, is analysed from data collected from the same population over an extended period of time, and in the current study these estimates overlapped well with the reversible catalytic model with a fixed SRR. This will allow more robust analyses of data with multiple survey points with a predictive element for SCR, though this model would benefit from testing against other datasets from areas of different endemicity and transmission patterns. However, the data suggest that the parsimonious reversible catalytic model (with the exploration of a possible change point) is robust when compared to more involved models and is appropriate for studies such as a single cross-sectional survey. At high transmission levels, the superinfection model would provide additional information. Ideally, a population with a broad age range should be used in all of these models, since this increases the accuracy of seroconversion estimates [31].

There are a number of caveats to be taken into account in interpreting these data. Firstly the serological assays for these surveys were conducted at different times and by different groups. However, the same ELISA protocol was used including the same recombinant protein as target antigen. Secondly, the five surveys were conducted in the same broad geographical area but in different villages and there is likely to be heterogeneity of transmission between the villages. The small sample sizes do not allow village specific estimates and so only overall SCRs are presented to represent population level exposure on a larger scale in a similar manner to PR. Additionally, surveys were conducted at different times of the year and this may have influenced serological profiles. However, as described above, some anti-malarial antibody responses have estimated half-lives of at least 10 years [3] and are therefore likely to be stable over shorter periods of time. This suggests that the seasonal timing of the survey should not affect the measurement of exposure, as shown in 2008/2009 in The Gambia [34], in contrast to point prevalence estimates of PR data. The 2011 survey included children only up to the age of 15 years and this may result in an underestimate of the overall SCR. However, this should not influence the observed trend in declining transmission significantly. Whilst these surveys were not originally conceived for longitudinal analyses the use of the same ELISA protocol and target protein construct, together with the correlation between the decrease in serological profile and decreases in the PR and U5MR over nearly 25 years suggests that these data are representative of the underlying disease dynamics in the study area.

## Conclusion

The rationale for this work is that in areas with low malaria transmission conventional measures of malaria transmission, such as the PR and the EIR, may have little discriminative power unless sampling of humans and mosquitoes is intense [1, 9, 35]. Serological metrics have promise as adjunct measures of transmission [6, 7] but for wider use and better understanding of the implications of these metrics, studies comparing serological outcomes with conventional biological and health measures (disease incidence, mortality, etc.) are required. This report documents a decline in anti-malarial specific antibodies in Farafenni over a time period of 23 years. This decline mirrors the decrease in other malariometric indices and of the U5MR in the Farafenni area [10, 12, 13] thereby confirming the suitability and validity of serology to monitor long-term changes in transmission. Anti-malarial antibody decay times in relation to age should be further explored, in order to obtain a more comprehensive insight into the immuno-epidemiology of (decreasing) malaria transmission.

## Additional file

10.1186/s12936-015-0939-1 Reverse catalytic models with a constant rate of seroconversion and multiple seroconversion rates.
