# Supplementary material for: Serology describes a profile of declining malaria transmission in Farafenni, The Gambia
Source: Malar J. 2015 Oct 22;14:416. doi: 10.1186/s12936-015-0939-1 (PMC4618886; doi:10.1186/s12936-015-0939-1)
Supplement: Supplementary file 1 — 10.1186/s12936-015-0939-1 Reverse catalytic models with a constant rate of seroconversion and multiple seroconversion rates. [file 12936_2015_939_MOESM1_ESM.docx]

**Supplementary Information**

***Constant rate of seroconversion***

Individuals are born seronegative. They become seropositive at rate$\lambda$, and then lose seropositivity at rate$\rho$. Let $S(t)$ be the probability of being seronegative at time$t$, and $I(t)=1-S(t)$ be the probability of being seropositive*.* $S$and $I$ change according to the following equations:

$$\begin{matrix} \frac{\partial S}{\partial t}=-\lambda S+\rho I \\ \frac{\partial I}{\partial t}=\lambda S-\rho I=\lambda-(\rho+\lambda)I \end{matrix}$$

With constant$\lambda$ since time$\tau$, this has the solution

$$I(t)=\left(I(\tau)-\frac{\lambda}{(\lambda+\rho)}\right)\mathrm{e}^{-(\lambda+\rho)(t-\tau)}+\frac{\lambda}{(\lambda+\rho)}, t>\tau$$

If $\lambda$ is constant since birth, then taking time $0$ as birth, $I(0)=0$ and at age $a$

$$I(a)=\frac{\lambda}{(\lambda+\rho)}\left(1-\mathrm{e}^{-(\lambda+\rho)a}\right)$$

***Multiple seroconversion rates***

Suppose there are $n$ time points $t_{1},...,t_{n}$ at which $\lambda$ changes, so that $\lambda$ is assumed to take $n+1$ values according to the time $t$: $\lambda_{1}$ before $t_{1}$, $\lambda_{2}$ between $t_{1}$ and $t_{2}$, $\lambda_{3}$ between $t_{2}$ and $t_{3}$, …, $\lambda_{n}$ between $t_{n-1}$ and $t_{n}$, and $\lambda_{n+1}$ after $t_{n}$. Defining$t_{0}=-\infty, t_{n+1}=+\infty$ , this can be summarised as $\lambda(t)=\lambda_{i}$ for$t_{i-1}<t\leq t_{i}$.

Suppose someone was born at time$b$, with$t_{j-1}<b\leq t_{j}$. Since $\lambda$ is piece-wise constant, the probability of being seropositive, which now depends on both $t$ and $b$, can be found by repeated use of equation :

$$\begin{matrix} I(t,b)=\frac{\lambda_{j}}{(\lambda_{j}+\rho)}\left(1-\mathrm{e}^{-(\lambda_{j}+\rho)(t-b)}\right) for b<t\leq t_{j} \\ I(t,b)=\left(I(t_{i-1},b)-\frac{\lambda_{i}}{(\lambda_{i}+\rho)}\right)\mathrm{e}^{-(\lambda_{i}+\rho)(t-t_{i-1})}+\frac{\lambda_{i}}{(\lambda_{i}+\rho)} for t_{i-1}<t\leq t_{i}, i=j+1,...,n+1 \end{matrix}$$

There may be multiple time points at which data are collected, $u_{1},...,u_{m}$. In the model as implemented, any set $u_{1}<u_{2}<...<u_{m}$ may be combined with any set of change points$t_{1}<t_{2}<...<t_{n}$. However $t_{n}$ needs to be before $u_{m}$ for there to be enough information to estimate all the parameters.

The model is fitted to data using a Bernoulli likelihood for each individual. The parameters estimated are $\lambda_{1},...,\lambda_{n+1}$ and$\rho$. Denote this set of parameters by$\theta$. Suppose that at time $u_{k}$ we have data from $N_{k}$ people, with people at each time indexed by$h$. Let $a_{kh}$ be each person’s age at the time the data were collected and $d_{kh}$ be $1$ if they are seropositive and $0$ if they are seronegative. The overall log-likelihood is given by:

where $I\left(.\right)$ is as defined in equation . This log-likelihood is maximised using the Stata maximum likelihood routines.

***Smoothed model***

We may want to estimate a large number of different values for $\lambda$ over time, in order to avoid specifying one or a few times when $\lambda$ might have changed. To prevent over-fitting the data, we use a penalised maximum likelihood approach. This penalises large variations in $\lambda$ by assuming that the log of the ratio of successive values of $\lambda$ follows a normal distribution with mean 0 and standard deviation $\sigma$, with $\sigma$ estimated from the data.

$\log(\lambda_{i+1}/\lambda_{i})\sim N(0,\sigma^{2})$for$i=1,...,n$

Hence the estimates of $\lambda$ over time are smoothed, unless the data strongly suggest a sudden change. The penalised log-likelihood is

where is the ordinary log-likelihood from equation .

***Stata program***

These models are implemented as a Stata program called revcat, which can fit both the simple reversible catalytic model and the smoothed model. It is freely available for download from the Statistical Software Components archive [26]. Within Stata, type "ssc describe revcat" to see a description, and "ssc install revcat" to install the program. Once it is installed, type "help revcat" to see a help file.
